# The attraction of evil. An investigation of factors explaining women’s romantic parasocial relationships with bad guys in movies and series

**DOI:** 10.3389/fpsyg.2024.1501809

**Published:** 2024-12-24

**Authors:** Holger Schramm, Annika Sartorius

**Affiliations:** Department of Media and Business Communication, Institute Human-Computer-Media, University of Würzburg, Würzburg, Germany

**Keywords:** romantic parasocial relationship, bad boys, women, retrospective imaginary involvement, masculinity

## Abstract

The attractiveness of bad boys can be seen as a cultural phenomenon that can be found in different areas of society and art. In the media, too, the bad boy fulfills social expectations in terms of masculinity and is often portrayed as dominant, violent, hard, unemotional and aggressive. Women may feel attracted to this male dominance under certain conditions. In order to investigate this phenomenon in the field of media psychology, this study examined women’s personality as predictor of romantic parasocial relationships (RPSR) with bad boys from movies or series, as well as feelings and experiences associated with such relationships. In an online questionnaire, 47 women were asked about specific personality traits, their RPSR with their favorite bad boy, and their perceived egocentric feelings and experiences associated with this RPSR. Both the love style ludus, which describes unattached playful love, and sensation seeking, which encompasses a woman’s inclination towards new experiences and adventures, emerge as predictors of an RPSR to a bad boy. Furthermore, it was found that a woman’s RPSR to a bad boy goes along with her retrospective imaginative involvement and sense of power. The findings show parallels to findings from social psychology that explain the attractiveness of bad boys in real relationships. Due to its correlative design, the study should be seen as a first step in the exploration of this media-psychological phenomenon, which will hopefully be followed by further studies with a stricter causality logic.

## 1 Introduction

The ambivalent character and his vagueness has established the bad boy in all kinds of media (Gopaldas and Molander, 2020). The bad boy is an attractive man with a conflicted personality characterized by dominant juveline masculinities, i.e., aggression, rebellion, and hypersexuality, and subordinate appealing qualities in the form of charisma, robustness, and sensitivity (Gopaldas and Molander, 2020). A particularly well-known example is the protagonist Christian Grey from the trilogy Fifty Shades of Grey, played by Jamie Dornan in the movie. As a media figure, the bad boy reflects social expectations of men through his dominant, violent, tough, and unemotional portrayal (Scharrer and Blackburn, 2018; Zeglin, 2016). Young adults in particular turn to the media to learn about appropriate and inappropriate behavior related to sexuality (see Erickson and Dal Cin, 2018; Harriger et al., 2021; Hust et al., 2008; Pinkleton et al., 2008), but find it difficult to categorize the behavior shown in films (Driesmans et al., 2016; Tukachinsky and Dorros, 2018). Men sometimes model themselves on dominant masculinity, while women may be attracted to it (Greenwood et al., 2021). But what effect does this attraction have on women who are confronted with bad boys through film and television? And which women are predisposed to be attracted to bad boys in terms of their personality?

## 2 Theory

### 2.1 Women’s romantic parasocial relationships with bad boys in movies and television

Horton and Wohl (1956) found that the feeling of intimacy was similar to that experienced in interpersonal orthosocial interactions in reality when interacting with media characters. This finding was the cornerstone of research into parasocial phenomena (Liebers and Schramm, 2023). For a long time, parasocial phenomena with a media character were defined exclusively as friendly, but both a heated argument and a love confession are orthosocial interactions, albeit emotionally polarized, and can be distinguished from each other on a parasocial level (Tukachinsky, 2010; Tukachinsky and Dorros, 2018). By definition, romantic parasocial relationships (RPSR) are long-term, cross-situational relationships with media figures that generate feelings of emotional and physical love, but are characterized by one-sidedness and do not include any reciprocal reactions (Liebers and Schramm, 2022). Adam and Sizemore (2013) also note that parasocial romantic attraction to a media figure resembles real-life affection except for the aspect of reciprocity.

While there are some empirical studies on parasocial relationships with antiheroes (see Bernhold, 2019; Brodie and Ingram, 2021; Dibble et al., 2023; Greenwood et al., 2021; Oliver et al., 2019), albeit not at the level of romantic attraction, few studies have been conducted on ambivalent characters such as the bad boy. Although the research on antiheroes shows that there is a prevalence of recipients for the villains, this is based on identification with the evil in the media persona. Although the bad boy is not a classic antihero because he also has a sensitive side, he can be compared to classic villains to a certain extent because of his malicious traits. In the following, previous studies on the phenomenon of romantically influenced parasocial phenomena, including those related to antiheroes, will be briefly outlined. Brodie and Ingram (2021) found a correlation between narcissism among recipients and the parasocial relationship with antiheroes. In addition to narcissism, a connection between romantic parasocial effects and Machiavellianism as well as psychopathy, i.e., the dark triad, was found (Liebers, 2021; Liebers and Schramm, 2022). The physical attractiveness of a character is also related to romantic parasocial attraction (Liebers and Schramm, 2017). In addition, Erickson and Dal Cin (2018) have suggested in their study on parasocial effects and their impact on the formation of romantic schemas and scripts that an RPSR can provide a safe space for testing romantic borderline experiences (Erickson and Dal Cin, 2018). Parasocial partners are “ubiquitously available and often easily and reliably accessible […]” (Hartmann, 2017, p. 134), while a romantic parasocial relationship can come very close to a real relationship in terms of the need for social closeness and relatedness (Adam and Sizemore, 2013).

Even though the bad boy combines characteristics of classic antiheroes, he is to be distinguished from them. Due to the limited amount of research on parasocial phenomena with ambivalent characters like the bad boy, the following section draws on studies on romantic relationships in reality that have found an attraction of female users to male dominance.

### 2.2 Attraction of male dominance

The attraction of male dominance, i.e., a woman’s desire to have a man at her side who is male, dominant, attractive (Labusch, 2019) and the toleration of immoral behavior as long as the hero character changes in the course of the plot (Gaddam et al., 2012), is already known from social psychology studies that have examined the attraction of offenders to women (cf. Giebel and Elbert, 2014; Isenberg and Isenberg, 1993; Labusch, 2019; Pfister, 2013). The focus of these studies was on women’s personality traits in relation to the question of whether these can predict a prevalence for the attractive effect of dominant men. A relationship with a bad boy offers a woman multiple opportunities to increase her self-esteem, which can be particularly appealing to women with narcissistic traits (Labusch, 2019). After all, a romantic relationship with a narcissist serves the purpose of increasing self-esteem (Wardetzki, 2011). People can also achieve increased self-esteem through helping others (Aronson et al., 2014). A woman’s tendency towards caring behavior therefore makes a man who is in need of help in multiple ways quite attractive (Labusch, 2019). This is also reflected in the motif of the good girl in films and series, who saves the bad boy from his plight through love and caring behavior (Gopaldas and Molander, 2020). Furthermore, a partner who promises freedom and adventure fits well into the love style ludus. Ludus describes wild, unattached, playful love (Lee, 1973). It is the style preferred by those who do not want a permanent partner but are looking for adventure in fleeting encounters. Consequently, it stands to reason that a woman who is attracted to male dominance prefers a high level of stimulation. The pursuit of new extraordinary experiences, such as encounters with nonconformist individuals, indicates a high level of lust for experience. Ultimately, a relationship characterized by passion and emotional ups and downs (Labusch, 2019) could be a very exciting experience for a woman with an average standard of living (Pfister, 2013).

If one follows the assumption that a bad boy offers a woman with a high urge to help the opportunity to live it out, an increase in self-esteem can be considered. Dealing with a criminal opens up many opportunities for a woman to feel supportive of herself, possibly even more so than she would feel in a real relationship (Liebers, 2021), since the criminal fits the criterion of a personality-disordered or addicted partner (Cermak, 1986) who needs to be saved. In terms of narcissism, there is also an obvious link to increased self-esteem (Wardetzki, 2011). In the study by Campbell et al. (2002), the constructs of narcissism and love style ludus correlate with each other, mediated by need for power and need for autonomy. Labusch (2019) also states in her work on the attractiveness of prisoners that high reflective control and a partner with high fate and behavioral control (Labusch, 2019) can increase a woman’s sense of power. Furthermore, people who approach relationships in a playful way and do not want to commit should, by logical extension, have a higher sense of autonomy. Campbell et al. (2002) describe the connection as follows: Those who show the least interest in a partnership and remain decidedly unattached have no obligations and a higher sense of freedom and autonomy.

### 2.3 Hypotheses

Young women are the main consumers of romantic narratives (Cohen, 1997; Ter Bogt et al., 2010) and may be attracted to male dominance (Labusch, 2019). Testing romantic relationships in a parasocial and thus “safe” environment (Cohen, 1997) is particularly important for women, as they are considered more “vulnerable” (Erickson and Dal Cin, 2018). This study therefore focuses on possible personality factors that may underlie a woman’s decision to get involved with a bad boy, as well as the egocentric feelings and experiences that may accompany such a relationship. The following hypotheses were derived: A relationship with a bad boy offers a woman multiple opportunities for self-esteem enhancement, which points to narcissistic traits as a fertile basis of a RPSR to a bad boy *(H1: narcissism)*. Furthermore, a RPSR to a bad boy promises short-term intense romantic experiences, which may be fueled by the need for sexual freedom in the form of the pronounced love style ludus *(H2: ludus)* and by the need for excitement and adventure *(H3: sensation seeking).* In addition, a romantic parasocial relationship with a needy bad boy should appear attractive to women with a strong helper urge (Labusch, 2019) *(H4: helper urge*).

The feeling of being the “only woman” in the bad boy’s life indicates an increased sense of self-esteem associated with RPSR *(H5: self-esteem).* At the same time, RPSR with a bad boy can go along with a feeling of superiority and power *(H6: power)* (Labusch, 2019). Women who approach relationships in a playful way and do not want to commit should also have a higher sense of autonomy through an RPSR (Campbell et al., 2002) *(H7: autonomy)*. Finally, an RPSR should enable retrospective imaginary involvement (RII) with the bad boy after reception *(H8: RII)*. RII allows variations of ideas related to the story to be mentally acted out (Slater et al., 2018). Although the concept of parasocial relationship and RII show certain similarities and correlate more strongly, the constructs are theoretically considered distinct (Liebers, 2021).

## 3 Method

Using an online questionnaire, women were asked about their potential romantic relationships with their favorite bad boy from a movie or series, as well as their egocentric feelings and experiences associated with this relationship and, finally, their personality, provided that they could specifically name such a favorite bad boy (the women were given a definition of what this means, displayed beforehand).

At the beginning of the questionnaire, the RPSR (11 items on a 5-point Likert scale, based on Liebers, 2021; *α* = 0.88) was assessed. With regard to the egocentric feelings and experiences, RII was measured using an adapted version of the original scale of Slater et al. (2018) (7 items on a 5-point Likert scale; α = 0.81). The items of the sense of autonomy subscale of Wirth et al. (2012) (3 items on a 5-point Likert scale; α = 0.73) were followed by the sense of power scale (6 items based on a 7-point Likert scale; based on Anderson et al., 2012; α = 0.81). Self-esteem was measured using the modified Rosenberg scale (6 items on a 4-point Likert scale; based on Ferring and Filipp, 1996; α = 0.88). With regard to the personality factors, sensation seeking was measured using the Sensation Seeking Scale (6 items based on a 5-point Likert scale; based on Zuckerman, 1984; α = 0.60). A subscale of the Love Attitudes Scale was used to measure ludus (4 items based on a 5-point Likert scale; based on Hendrick et al., 1998; α = 0.78). The subscale “agape” of the Marburg Love Style Inventory (MEIL) was selected to measure the urge to help (6 items based on a 5-point Likert scale; based on Bierhoff et al., 1993; α = 0.77). Finally, narcissism was measured using the corresponding subscale of the Dark Triad Scale (7 items using a 5-point Likert scale; based on Jones and Paulhus, 2014; α = 0.81).

The final sample consisted of 47 women with an average age of *M* = 23.55 years. The majority of the women are heterosexual, with 11 women (23.4%) stating that they were bisexual. Individuals who, in contravention of the restrictions on participation, stated that they were male, and one person who provided an impossible age, were excluded from the survey. Women who did not know of any media bad boys were also excluded.

To determine the appropriate sample size for the study, an *a priori* power analysis was conducted using G*Power (power = 0.80; alpha error probability = 0.05). Based on the cited literature and studies, effects of at least medium strength were to be expected. Instead of including all factors simultaneously in a multiple regression, we found it more appropriate to calculate separate simple regressions. This decision was not made for statistical reasons, such as to avoid multicollinearity, but for theoretical reasons, since the personality factors, especially ludus and sensation seeking, overlap to some extent in terms of their theoretical concept, and multiple regression counteracts this overlap due to its statistical requirements. Under these conditions, G*Power calculated that at least *N* = 25 is required for the lower limit of a large effect size of *f^2^* = 0.35 and *N* = 55 for the lower limit of a medium effect size of *f^2^* = 0.15. With a sample size of *N* = 47, we are well within the target range.

## 4 Results

Since we were interested in the isolated explanatory power of each individual personality trait, the hypotheses were tested using simple regression analyses. Contrary to expectations, narcissism (*M* = 2.64, *SD* = 0.85) could not be confirmed as a predictor *(H1)* of RPSR (*M* = 3.08, *SD* = 0.78), *F(*1, 45) = 0.70, *p* = 0.41. The love style ludus (*M* = 1.71, *SD* = 0.85), on the other hand, was significant as a predictor *(H2)* of RPSR, *F*(1, 45) = 8.05, *p* < 0.01 (*R^2^* = 0.13), although the average intensity of this love style was not particularly high in the sample. Likewise, sensation seeking (*M* = 3.0, *SD* = 0.74) was a significant predictor *(H3)* of RPSR, *F*(1, 45) = 4.23, *p* < 0.05 (*R^2^* = 0.07), whereas a woman’s urge to help (*M* = 3.95, *SD* = 0.65) *(H4)* was not significant, *F*(1, 45) = 2.23, *p* = 0.14, although the average intensity of this factor was particularly high in the sample.

**Figure 1:**
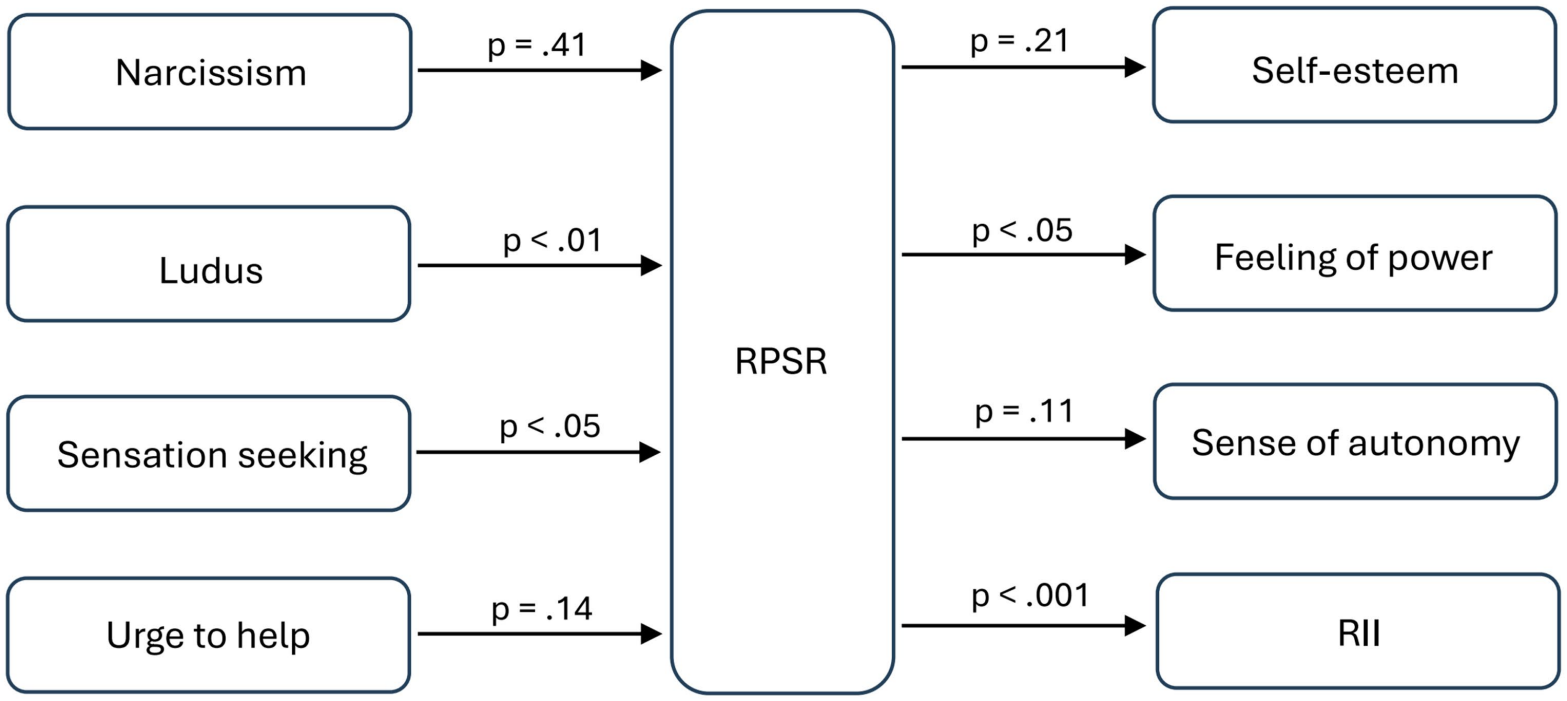
Overview of tested connections.

Furthermore, the increased self-esteem (*M* = 3.1, *SD* = 0.65) as a possible consequence of a RPSR (*H5*) could not be confirmed, *F*(1, 45) = 1.62, *p* = 0.21. However, RPSR can significantly predict the feeling of power (*M* = 3.49, *SD* = 1.23) (*H6*), *F*(1, 45) = 6.75, *p* < 0.05 (*R^2^* = 0.11). A stronger sense of autonomy (*M* = 3.43, *SD* = 0.99) as a side effect of a RPSR of a woman to a bad boy (*H7*) could not be confirmed, *F*(1, 45) = 2.64, *p* = 0.11. Finally, RPSR predicts RII (*M* = 2.7, *SD* = 0.97) in a significant manner *(H8)*, *F*(1, 45) = 13.88, *p* < 0.001, and with relatively high variance explanation (*R^2^* = 0.22). Figure 1 shows all the connections tested again at a glance.

## 5 Discussion

Women may be attracted to dominant masculinity, as embodied by bad boy characters that are widely represented in the media (cf. Giebel and Elbert, 2014; Isenberg and Isenberg, 1993; Labusch, 2019; Pfister, 2013). This study examined this phenomenon in the form of RPSR as well as the individual basis (in the form of personality traits) and egocentric feelings and experiences that may be associated with such RPSR. It was found that women’s ludus and sensation seeking can predict RPSR with a bad boy. In addition, the results suggest that RPSR can affect both RII and the sense of power after reception.

The reason for the lack of significance of narcissism could be attributed to the sensitive side of the bad boy, i.e., the appealing qualities, since these could be interpreted as weakness, which is rejected by women with pronounced narcissism (Wardetzki, 2011). A narcissistic woman prefers a partner who is just as narcissistic as she is. Since the bad boy only complements the narcissistic woman to a limited extent due to his sensitive side, she may reject him due to a lack of identification. That identification could be relevant as a construct in this context is supported by studies that found a connection between identification with an antihero and liking of that character (cf. Dibble and Rosaen, 2011; Oliver et al., 2019). The urge to help is also not a predictor of RPSR. A woman can only imagine helping in an RPSR and not actually take action. It stands to reason that the mere desire to help, without actually doing so, is not enough to demonstrate an effect.

Since the increase in self-esteem was assumed to be the result of a satisfied need for help and the strong expression of narcissistic personality traits, and since these did not prove to be significant, the result of a non-significant increase in self-esteem fits into the overall picture. The assumption that a woman who likes to remain unattached and is looking for adventures in the context of relationships has an increased sense of autonomy as a result of an RPSR was not confirmed, which is surprising, however, since ludus and sensation seeking significantly predicted the RPSR and an increased sense of autonomy should have been the plausible consequence.

The limitations of this study lie primarily in its correlative design. However, this type of retrospective survey design without experimental manipulation has been used for decades (Liebers and Schramm, 2019) and is still used primarily to research parasocial relationships (Schramm et al., 2024). In this respect, this study was also based exclusively on the memory of the subjects, since no stimulus material was used. As a result, the subjects had little opportunity to test the consistency of our definition and their idea of a bad guy. A selection of scenes and protagonists for control purposes would create transparency and support the validity of the results. The sample size should be seen as a further limitation: because it was incredibly difficult to find women who were willing to talk about their potential romantic relationships with their favorite bad boy and their love style “ludus,” we settled for a sample size of *N* = 47—it was hard-won, but in view of possible smaller effects it was perhaps a little underpowered. Further limitations of this study are evident in the measurement instruments. The measurement of sensation seeking was insufficient with a Cronbach’s 𝛼 = 0.60. The sense of autonomy was measured a little too narrowly and economically with three items—a broader measurement on a broader sample in a follow-up study could possibly show the hypothetically assumed effect after all.

Despite the limitations, this first study on this topic clearly shows the potential of media psychological research on media-transmitted bad boys as embodiments of male dominance and their effect on women. Since half of the hypotheses were confirmed, the findings as a whole do indicate that the phenomenon of male dominance attracting specific women can be transferred to the media context. Possible future studies could now take a closer look at the effects of bad boys on certain women and clearly demonstrate these effects in experimental settings. Of particular interest, however, are the long-term effects that parasocial interaction with such bad boys can have on the behavior of young women. Especially, the RII showed great potential. The RII indicates a strong influence of the bad boy narrative and enables a distinction to be made between the peri-receptively experienced and post-receptively imagined narrative, which could become the focus of follow-up studies.

## Data Availability

The raw data supporting the conclusions of this article will be made available by the authors, without undue reservation.
